# Unusual synchronous skeletal muscle and lung metastasis in papillary thyroid cancer: A case report and review of the literature

**DOI:** 10.3892/ol.2014.2742

**Published:** 2014-11-27

**Authors:** JUN YANG, LIN-FA LI, XIU-MING ZHANG, QIN XU, JUN ZHANG, WAN-WEN WENG, MENG-JIE DONG

**Affiliations:** 1 Department of Nuclear Medicine, The First Affiliated Hospital, College of Medicine, Zhejiang University, Hangzhou, Zhejiang 310003, P.R. China; 2 Department of Pathology, The First Affiliated Hospital, College of Medicine, Zhejiang University, Hangzhou, Zhejiang 310003, P.R. China; 3 Positron Emission Tomography Center, The First Affiliated Hospital, College of Medicine, Zhejiang University, Hangzhou, Zhejiang 310003, P.R. China

**Keywords:** papillary thyroid cancer, skeletal muscle metastasis, lung metastasis

## Abstract

Papillary thyroid cancer (PTC) frequently metastasizes to the cervical lymph region and less often to the lung and bone. Metastasis to the skeletal muscles from PTC is extremely rare, especially concurrent lung and skeletal muscle metastases. The present study reports the case of a 31-year-old man with synchronous metastasis to the skeletal muscle and lung from PTC, six years following total thyroidectomy and consecutive ^131^Iodine treatments. Magnetic resonance imaging (MRI) revealed a 1.7×1.2×1.5 cm mass in the left gastrocnemius muscle, indicating a neurogenic tumor. The mass was subsequently resected and confirmed via histopathology to be metastatic PTC. We propose that, in the follow-up of patients with PTC, the measurable serum thyroglobulin level, whole body scan and other imaging modalities including MRI or positron emission tomography/computed tomography, must be closely monitored for potential distant metastases, particularly in cases of PTC with aggressive pathological behavior.

## Introduction

The majority of thyroid cancers (90%) are differentiated thyroid cancers (DTC), a term which includes both papillary and follicular cancer (1). Thyroid carcinoma accounts for ~1% of all new malignant disease and ~0.5 and 1.5% of cancers in males and females, respectively (2). DTC progression is generally slow, with a low grade malignancy and excellent long-term survival rates. The overall 10-year survival rate of DTC is >90% (3). Among DTC, the incidence of papillary thyroid cancer (PTC) was 7.9 per 100,000 individuals, the mortality rate was ~0.4 per 100,000 individuals and the overall survival rate was 98.3% (4,5). Patients with DTC respond to total thyroidectomy, radioiodine ablation and levothyroxine suppression therapy. Distant metastases occur during follow-up in 2.2–23% of patients (6–8), and are usually identified in the lung and bone. Less frequently, they are detected in the brain, liver or other sites, and indicate a significantly poorer prognosis (9,10). However, metastasis to the skeletal muscles from PTC is extremely rare, particularly synchronous lung and skeletal muscle metastases (11,12). The present study reports the case of a 31-year-old male with PTC and concurrent metastasis to the left gastrocnemius muscle and lungs. Written informed consent was obtained from the patient.

## Case report

**Figure 1:**
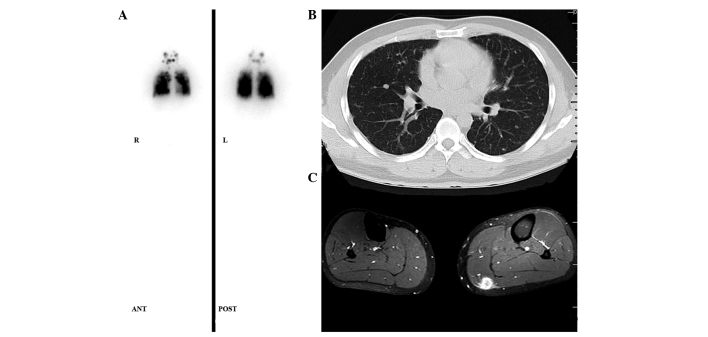
(A) ^131^Iodine whole-body scan, seven days after an oral therapeutic dose of 3700 MBq ^131^Iodine in 2007, showed a diffuse uptake in the chest and focus of the neck. (B) Computed tomography scan of the thorax showed extensive metastatic disease of the lungs. (C) Magnetic resonance imaging showed gastrocnemius muscle metastasis in the left leg.

In May 2007, a 31-year-old Chinese male presented to the Department of Surgical Oncology with palpable nodules of the thyroid in May 2007. An ultrasound (US) of the patient’s neck revealed hypoechoic multinodules with microcalcification in the bilateral thyroid, the largest of which was located in the left lobe (~3.0 cm). Cervical lymph node enlargement was also observed with microcalcification. Thyroid function tests indicated a euthyroid state. The patient had no family history of thyroid disease and denied any exposure to external or accidental radiation. Serum thyroglobulin (Tg) level was measured at 82.2 ng/ml (normal range, <55 ng/ml), with negative anti-Tg antibodies (TgAb). Following total thyroidectomy and central cervical node dissection, the patient was diagnosed with laryngotracheal invasion from PTC (pathological stage T4a N1a M1pul, 2010 AJCC) (13). The patient subsequently received radioiodine treatment four times (total dose, 24050 MBq). A whole body scan (WBS) revealed ^131^Iodine uptake in the lung (Fig. 1A), consistent with visible lesions on a computed tomography (CT) scan (Fig. 1B), which indicated pulmonary metastases.

**Figure 2:**
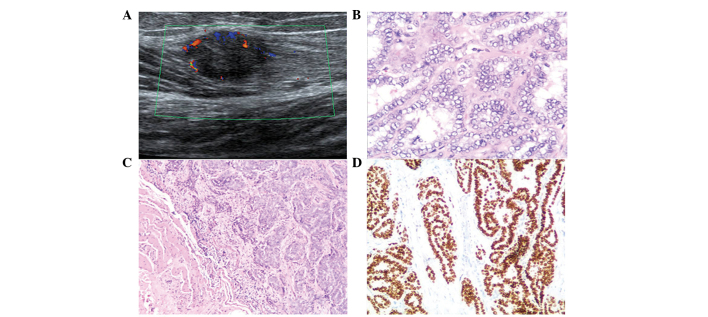
(A) Doppler ultrasound revealed a hypoechoic lesion with peripheral hypervascularity. (B) Infiltrating clusters of papillary tumor cells in the skeletal muscle (HE staining; magnification, ×50). (C) Typical nuclear inclusions and grooves (HE staining; magnification, ×200). (D) Immunohistochemistry showed tumor cells positive for thyroid transcription factor 1. HE, hematoxylin and eosin.

The patient was referred to the First Affiliated Hospital (Hangzhou, China) in July 2013, with a three-month history of a slowly increasing mass in the left gastrocnemius muscle. On physical examination, the patient’s vital signs were stable, and a soft mass of ~1.5 cm in size was palpable in the left leg with no inflammatory surface. Serum Tg level was 80.2 ng/ml (normal range, <1 ng/ml). However, TgAb level was not available during this time. The results of tests for other biochemical tumor markers, including α-fetoprotein (2.8 ng/ml; normal range, <20.0 ng/ml), carcino-embryonic antigen (1.9 ng/ml; normal range, <5.0 ng/ml), sugar antigen 199 (4.2 U/ml; normal range, <37 U/ml), sugar antigen 125 (13.0 U/ml; normal range, <35 U/ml) and total prostate specific antigen (2.103 ng/ml; normal range, <4.0 ng/ml), were within normal limits. Magnetic resonance imaging (MRI) revealed a soft-tissue mass that was markedly low in signal intensity on T1-weighted images, and enhanced after administration of contrast material (Fig. 1C). Doppler US showed a 1.7×1.2×1.5 cm solitary, hypoechoic nodule with peripheral hypervascularity (Fig. 2A). A diagnosis of neurogenic tumor was highly suspected due to the morphological features of the mass. A local marginal resection was subsequently performed. Histopathology revealed the mass to be a metastatic papillary tumor (Fig. 2B and C), and immunohistochemical examination showed that the cells were positive for thyroid transcription factor-1 (TTF-1) (Fig. 2D), indicating gastrocnemius muscle metastasis from PTC. Concurrently, CT imaging of the chest revealed innumerable, moderately well-circumscribed nodules in the lung with high ^131^Iodine uptake, indicating tumor metastases. After surgery, the patient was treated with ^131^Iodine (6660 MBq). The patient’s Tg levels decreased to 40.6 ng/ml (normal range, <1 ng/ml) and pulmonary metastases were stable after six months of follow-up.

## Discussion

Papillary thyroid cancer is commonly associated with lymphatic spread to regional lymph nodes. Distant metastases occur more rarely, usually involving the lungs and bone (1–7% of patients) (14), but occasionally occurs in the brain, sphenoid sinus, orbit, adrenal, kidney and ovary (15–18). A retrospective review of the literature revealed only two case reports of muscle metastases arising from PTC (11,12). It has been hypothesized that skeletal muscle is a hostile environment for proliferating cancer cells, due to muscle motion and unadapted muscle pH (19), which may explain the lack of such cases. Synchronous lung and skeletal muscle disease from PTC is an extremely rare manifestation (18). To the best of our knowledge, only two cases have been previously reported (Table I). Bruglia *et al* (11) reported the case of a 44-year-old male with PTC, with a poorly differentiated thyroid carcinoma component and metastases to the thigh muscle, skin, lung, mediastinum and brain. The aggressiveness of the tumor led to mortality eight years following total thyroidectomy. Luo *et al* (12) reported the case of a 29-year-old male patient who had unusual metastasis sites in the lung, kidney and erector spinae from PTC. The authors concluded that suspected tumors must be considered as potential metastases from thyroid carcinomas in the clinical setting. The current study reports a rare case of PTC associated with diffuse metastases to the lung and gastrocnemius muscle, six years following total thyroidectomy and radioiodine treatment.

The particularly good prognosis and long-term survival of PTC patients are significantly reduced in those with distant metastasis (DM). The early differential diagnosis of distant metastases of thyroid carcinoma remains difficult using common diagnostic modalities such as US, Tg levels and ^131^I-WBS. Although postoperative serum Tg levels and ^131^I-WBS scans are sensitive methods in the detection of metastatic disease, accurately localizing the source of the abnormalities can be problematic, particularly in soft tissues. In addition, radioiodine-negative thyroid cancer may account for ≤20% of cases of DTC, which presents challenges in the localization of metastatic lesions, even if the serum Tg level is evaluated (20). Other imaging modalities, including MRI and 2-deoxy-2-(^18^F)fluoro-D-glucose (^18^FDG)-positron emission tomography (PET), may also be valuable in the follow-up of thyroid cancer metastases, particularly in patients with elevated Tg levels and normal radioiodine WBS (21).

Various factors may contribute to the development of DM, including large and multifocal primary tumors, extrathyroidal extension, aggressive histology and advanced age (>45 years) (22). Due to the rarity of synchronous lung and skeletal muscle metastasis of PTC, characteristic risk factors have not yet been identified. In the present case, postoperative pathology revealed that DTC had invaded the laryngotracheal region. Microscopic or gross invasion of the tumor into the perithyroid, aggressive histology or vascular invasion may be associated with an intermediate-high risk of recurrence (23). Mortality is increased in patients with distant metastases, particularly in those aged >45 years (6). Notably, the patient in the present study was only 37 years of age, and remains alive six years following total thyroidectomy and radioiodine treatment, which may support this observation.

In conclusion, this study presents a rare case of synchronous skeletal muscle and lung metastasis from PTC. We propose that Tg measurement, WBS and other imaging modalities, including US, MRI and ^18^FDG-PET, must be utilized during follow-up of patients with PTC, to detect possible uncommon distant metastases, particularly in cases of PTC that exhibit aggressive pathological behavior.

## Figures and Tables

**Table I:** Synchronous lung and skeletal muscle metastases from PTC reported previously in the literature.

Author	Age, y/gender	Histology	TNM classification after operation	Extrathyroid extension	Site of metastasis	Survival after diagnosis of metastasis
Bruglia *et al* (11)	44/male	PTC	T3N1bM0	Yes	Biceps femoris, lung, skin, brain	7 years
Luo *et al* (12)	29/male	PTC	TXN1M1	NA	Erector spinae, lung, kidney	-
Present case	31/male	PTC	T4N1aM1	Yes	Gastrocnemius muscle, lung	Alive

NA, not available; PTC, papillary thyroid carcinoma; TX, Tumor size was not available; y, years.
